# Supplementary material for: Rats exhibit similar biases in foraging and intertemporal choice tasks
Source: eLife. 2019 Sep 18;8:e48429. doi: 10.7554/eLife.48429 (PMC6794087; doi:10.7554/eLife.48429)
Supplement: Transparent reporting form [file elife-48429-transrepform.docx]

***eLife’s* transparent reporting form**

We encourage authors to provide detailed information *within their submission* to facilitate the interpretation and replication of experiments. Authors can upload supporting documentation to indicate the use of appropriate reporting guidelines for health-related research (see [EQUATOR Network](http://www.equator-network.org/%20)), life science research (see the [BioSharing Information Resource](https://biosharing.org/)), or the [ARRIVE guidelines](http://www.plosbiology.org/article/info:doi/10.1371/journal.pbio.1000412) for reporting work involving animal research. Where applicable, authors should refer to any relevant reporting standards documents in this form.

If you have any questions, please consult our Journal Policies and/or contact us: [editorial@elifesciences.org](mailto:editorial@elifesciences.org).

**Sample-size estimation**

- You should state whether an appropriate sample size was computed when the study was being designed
- You should state the statistical method of sample size computation and any required assumptions
- If no explicit power analysis was used, you should describe how you decided what sample (replicate) size (number) to use

Please outline where this information can be found within the submission (e.g., sections or figure legends), or explain why this information doesn’t apply to your submission:

The number of rats per experiment and number of observations per rat were chosen based on our prior published study using the same foraging task (Kane et al., 2017). A power analysis was conducted using data from a similar experiment from that paper, using the “simr” package in R (Green & MacLeod, 2016, *Methods in Ecology and Evolution*). This package uses Monte Carlo simulation to estimate power for linear and generalized linear mixed effects models. This analysis revealed that power > 0.95 in our prior study, and we have similar effect sizes and sample sizes in the present experiments.

Both the number of rats and number of observations per rat is mentioned throughout the results section, accompanying statistical analysis for each experiment.

**Replicates**

- You should report how often each experiment was performed
- You should include a definition of biological versus technical replication
- The data obtained should be provided and sufficient information should be provided to indicate the number of independent biological and/or technical replicates
- If you encountered any outliers, you should describe how these were handled
- Criteria for exclusion/inclusion of data should be clearly stated
- High-throughput sequence data should be uploaded before submission, with a private link for reviewers provided (these are available from both GEO and ArrayExpress)

Please outline where this information can be found within the submission (e.g., sections or figure legends), or explain why this information doesn’t apply to your submission:

Each experiment was conducted only once. All collected data was included for analysis (no data were considered outliers).

**Statistical reporting**

- Statistical analysis methods should be described and justified
- Raw data should be presented in figures whenever informative to do so (typically when N per group is less than 10)
- For each experiment, you should identify the statistical tests used, exact values of N, definitions of center, methods of multiple test correction, and dispersion and precision measures (e.g., mean, median, SD, SEM, confidence intervals; and, for the major substantive results, a measure of effect size (e.g., Pearson's r, Cohen's d)
- Report exact p-values wherever possible alongside the summary statistics and 95% confidence intervals. These should be reported for all key questions and not only when the p-value is less than 0.05.

Please outline where this information can be found within the submission (e.g., sections or figure legends), or explain why this information doesn’t apply to your submission:

Details regarding mixed effects models of behavioral data are briefly discussed in the results prior to reporting of these analyses (primarily in paragraphs starting on lines 153 and 268). Full details for each of the models, including the formula in the syntax of the software package used to fit the models is in the Methods (lines 528 and 566). Exact p-values are reported when p > .001, otherwise they are reported as p < .001. Full details for each of the quantitative models of cognitive biases can be found primarily in the Methods (sections starting on line 573 and 662).

(For large datasets, or papers with a very large number of statistical tests, you may upload a single table file with tests, Ns, etc., with reference to sections in the manuscript.)

**Group allocation**

- Indicate how samples were allocated into experimental groups (in the case of clinical studies, please specify allocation to treatment method); if randomization was used, please also state if restricted randomization was applied
- Indicate if masking was used during group allocation, data collection and/or data analysis

Please outline where this information can be found within the submission (e.g., sections or figure legends), or explain why this information doesn’t apply to your submission:

All experiments were tested within subjects, there was no group allocation.

**Additional data files (“source data”)**

- We encourage you to upload relevant additional data files, such as numerical data that are represented as a graph in a figure, or as a summary table
- Where provided, these should be in the most useful format, and they can be uploaded as “Source data” files linked to a main figure or table
- Include model definition files including the full list of parameters used
- Include code used for data analysis (e.g., R, MatLab)
- Avoid stating that data files are “available upon request”

Please indicate the figures or tables for which source data files have been provided:

Trial-by-trial data for each rat from each experiment are included as source files. Data from all foraging experiments are in the ‘forageDat.csv’ file and intertemporal choice data in the ‘itcDat.csv’ file.

Code to fit mixed effects models to the foraging data are found in ‘forage_lme_eLife.R’, and code to fit mixed effects models to intertemporal choice data in ‘itc_lme_eLife.R’.
